# Innovations in peritoneal dialysis fluid: biocompatible formulations and expanded therapeutic applications

**DOI:** 10.1080/0886022X.2025.2583626

**Published:** 2025-11-24

**Authors:** Qing Xu, Zhifeng Zhou, Lu Jin, Chen Liu, Peiyun Li, Fang Wang, Ling Zhang, Ping Fu

**Affiliations:** Department of Nephrology, Kidney Research Institute, West China Hospital of Sichuan University, Chengdu, China

**Keywords:** Peritoneal dialysis, peritoneal dialysis fluid, biocompatibility, l-Carnitine, hyperbranched polyglycerols

## Abstract

Peritoneal dialysis (PD) is a critical renal replacement therapy for end-stage kidney disease. However, its adoption remains limited, partly due to peritoneal fibrosis and metabolic complications induced by conventional glucose-based peritoneal dialysis fluid (PDF), the primary complications in PD patients and the leading cause of technique failure. This review outlines three innovative approaches to overcome these limitations: (1) novel biocompatible osmotic agents (L-carnitine can improve the metabolism of the peritoneum, and hyperbranched polyglycerol provides sustained ultrafiltration with dual peritoneal/renal protection), (2) advanced biocompatible buffers (citrate and pyruvate), and (3) peritoneal protectants (glycosaminoglycans and molecular hydrogen) that collectively mitigate fibrosis while protecting membrane function. These developments indicate a future direction for biocompatible PDF: integrating dual or triple protective functions of “osmotic agents + buffers + additives” synergy—optimizing hybrid formulations and accelerating clinical translation. Furthermore, we explored the emerging role of PD in refractory heart failure, where specialized PDF and steady-concentration protocols enhanced ultrafiltration efficiency. These advances address volume overload in cardiorenal syndrome, expanding PD’s therapeutic scope of PD to systemic conditions such as heart failure.

## 1. Introduction

Peritoneal dialysis (PD) is a critical home-based renal replacement therapy (RRT) for patients with end-stage kidney disease (ESKD). The treatment modality selection is significantly influenced by national healthcare policies and dialysis infrastructure [1]. In 2018, approximately 11% of maintenance dialysis patients received PD, with more than half of these cases concentrated in China, Mexico, the USA and the Thailand [2]. Additionally, the use of PD has grown rapidly in several regions including the United States, China, and Thailand. In the context of the increasing prevalence of ESKD and constrained healthcare resources, PD is a resource-efficient alternative [1].

The peritoneal dialysis fluids (PDFs) contain osmotic agents, buffers and electrolytes. Osmotic agents generate the osmotic pressure necessary for uremic toxin and fluid removal in ESKD patients, while buffers maintain acid-base balance and correct electrolyte disturbances [3].

Currently, there are four types of available PDFs: glucose-based PDF, pH-neutral, low-glucose degradation products (GDPs) PDF, amino acid PDF, and icodextrin PDF. Among these, glucose PDFs remain predominant. However, high glucose and GDPs levels induce peritoneal inflammation, oxidative stress, hyperglycemia and insulin resistance. This is supported by a recent systematic review and meta-analysis by Goossen et al. [4]. Moreover, this review found that although icodextrin PDF can significantly reduce peritoneal glucose absorption as a more biocompatible alternative, it can cause hypermaltesemia. Furthermore, the strategy of a single overnight icodextrin dwell may thus be inadequate for reducing the glycemic load, underscoring the need to develop other biocompatible PDFs.

**Figure 1.**
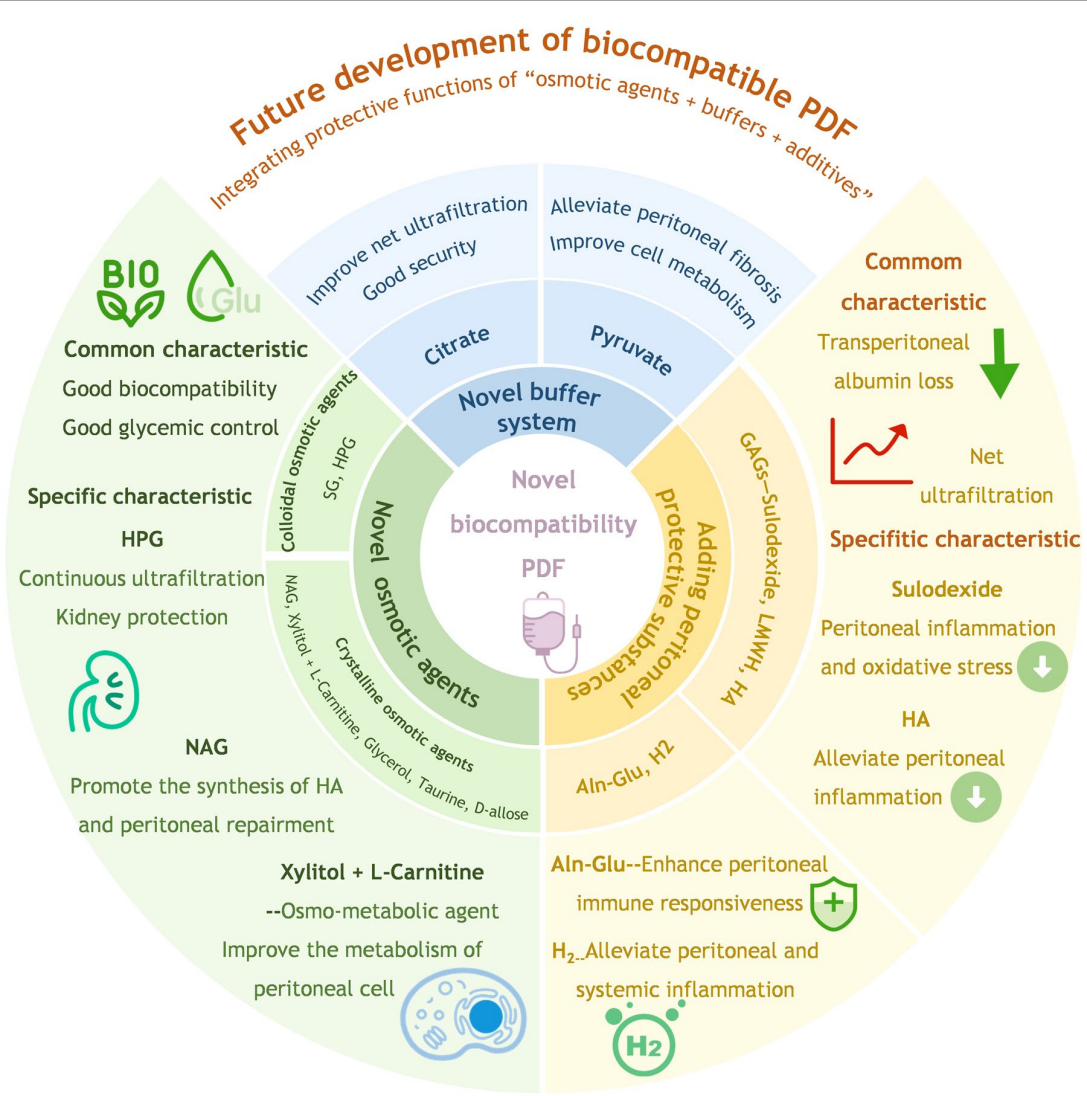
Current progress in novel biocompatible peritoneal dialysis fluid (PDF).

This review examines the advances in biocompatible PDF through three dimensions: innovative osmotic agents, advanced buffer systems, and peritoneal protective additives. And we proposed future development direction of biocompatible PDF: integrating dual or triple protective functions of ‘osmotic agents + buffers + additives’ synergy—optimizing hybrid formulations and accelerating clinical translation (Figure 1). Furthermore, we explored the application of a novel PDF for solute clearance in heart failure management.

Although this review covers a wide range of innovative PDFs, it is crucial to recognize that most of the novel formulations are still in experimental stage. The translation into clinical practice remains a considerable journey ahead, underscoring both the potential and the challenges in the field.

## 2. Methods

This narrative review was conducted to comprehensively summarize the current landscape of innovations in biocompatible peritoneal dialysis fluids. A systematic literature search was performed across 3 electronic databases, including PubMed, Embase, and Scopus, for articles published up to October 1st, 2025. The detailed information of search strategy is in Additional file 1.

The initial literature search and the removal of duplicates were performed independently by two authors (Q.X. and Z.Z.). Subsequently, the two authors (Q.X. and Z.Z.) independently screened the titles and abstracts of the retrieved records to identify studies relevant to this review. Full-text articles of potentially eligible studies were then assessed by the same two authors against pre-defined inclusion criteria: studies focusing on novel PDF formulations and expanded applications (e.g. in heart failure). Any discrepancies regarding study inclusion were resolved by consultation with a third author (L.J.).

## 3. Traditional peritoneal dialysis fluid

### 3.1. Glucose peritoneal dialysis fluid

Glucose PDFs utilize glucose as an osmotic agent and lactate as a buffer. Glucose remains the predominant osmotic agent clinically owing to its proven osmotic efficacy, cost-effectiveness, rapid metabolism, and dual role as both an osmotic agent and a physiological energy source. However, this formulation presents several inherent limitations, including a high glucose concentration, hyperosmolality, and acidic pH (typically 4.0–5.5). These characteristics contribute to peritoneal membrane injury (manifested as inflammation, fibrosis, and neovascularization) and systemic metabolic complications (including hyperglycemia, insulin resistance, and increased cardiovascular risk) during long-term therapy [5–7].

GDPs form during high-temperature sterilization of PDF. Exposure to GDPs damages both peritoneal mesothelial and vascular endothelial cells [8,9]. Moreover, GDPs drive the formation of advanced glycation end products (AGEs), which further exacerbate peritoneal injury [10]. To minimize the generation of GDPs, a conventional acidic dialysate (pH 4.5–5.5) was developed. However, a low pH induces peritoneal damage and infusion-related pain.

High glucose levels, GDPs, and AGEs in PDF are key sources of biological incompatibility. They induce peritoneal mesothelial cells to overproduce extracellular matrix components and upregulate profibrotic mediators such as transforming growth factor-beta (TGF-β) and plasminogen activator inhibitor-1. In addition, these compounds activate protein kinase C, NADPH oxidase, and mitochondrial metabolism, leading to excessive reactive oxygen species generation [11,12]. Collectively, these pathways promote mesothelial-mesenchymal transition, oxidative stress, and inflammation, resulting in peritoneal structural and functional alterations, fibrosis, UF failure, and an increased risk of cardiovascular mortality risk [13,14].

### 3.2. pH-neutral, low-glucose degradation product (GDP) peritoneal dialysis fluid

A dual-chamber bag system was developed to reduce the formation of GDPs. This system separates glucose and buffer components during sterilization [15], maintaining concentrated glucose at low pH (2.8–3.2) to minimize GDPs formation. Upon mixing, the PDF achieved physiological pH (7.0–7.4), significantly enhancing biocompatibility compared to glucose PDF. In addition, neutral, low-GDP PDF can better preserve residual kidney function and maintains urine output [16].

Traditional PDF formulations are restricted to lactate buffers because bicarbonate tends to react with calcium and magnesium, forming precipitates. Dual-chamber packaging circumvents this limitation, enabling bicarbonate buffer utilization. While this innovation addresses pH-related issues, it still relies on high glucose concentrations for osmotic drive, failing to resolve glucose-associated metabolic complications and peritoneal injury [17–19].

### 3.3. Amino acid peritoneal dialysis fluid

Amino acid PDF utilizes 1.1% of amino acids as an osmotic agent with lactate buffers. Low GDP content contributes to the preservation of peritoneal morphological integrity and UF function. Furthermore, owing to their low molecular weight, amino acids can be efficiently absorbed across the peritoneum, thereby improving the nutritional status of patients [20]. However, limitations include the transient UF duration and the risk of metabolic acidosis with prolonged use. High-dose administration may cause significant gastrointestinal side effects (including appetite suppression) and increased nitrogen load.

### 3.4. Icodextrin peritoneal dialysis fluid

Icodextrin is a glucose polymer (13–19 kDa) that exerts osmotic effects through colloidal osmolality. This PDF enables the maintenance of osmolality and sustained UF [21–23]. Clinically, icodextrin substitution reduces insulin resistance, improves lipid profiles, and lowers cardiovascular risk [24–27]. A clinical study confirmed better peritoneal integrity preservation, reduced vascular endothelial injury, and attenuated fibrosis compared with glucose PDF [28,29].

Metabolized to maltose, icodextrin carries the risk of maltose accumulation during extended use (particularly in hepatic impairment). In addition, glucometers using the glucose dehydrogenase pyrroloquinoline quinone (GDH-PQQ) method can yield falsely high blood glucose readings in the presence of maltose [30].

Although icodextrin and amino acids demonstrate superior biocompatibility to PDF, their limitations preclude complete substitution [31–33]. To address these constraints, researchers have focused on three approaches: novel osmotic agents, novel buffers, and peritoneal protective additives, collectively aiming to create biocompatible PDF with enhanced clinical benefits.

## 4. Innovations in osmotic agents- crystalline osmotic agent

### 4.1. N-acetylglucosamine (NAG)

*NAG* (221 Da) exhibits potent anti-inflammatory properties [34]. A study in uremic rats with peritonitis demonstrated that NAG significantly reduces intraperitoneal and systemic inflammation [35]. Ciszekwicz et al. confirmed that NAG has superior biocompatibility compared to glucose: long-term exposure induces neither oxidative stress nor inflammation/fibrosis in mesothelial cells [34].

During dialysis, glycemia and insulin levels were significantly lower in rats treated with NAG than in those treated with the glucose PDF. In addition, researchers observed that NGA can improve UF and decrease the permeability of the peritoneum to proteins [36]. The possible reasons for the improvement in UF and permeability are explained in the study by Ciszewicz et al. [34]. They found that NAG can promote the synthesis of hyaluronic acid (HA), which can exert anti-inflammatory effect and enhance the negative charge barrier of the peritoneum. Long-term rat studies corroborate these findings: NAG can reduce inflammation and stimulate HA production [37].

### 4.2. L-carnitine

*L-carnitine* (161.2 Da) is a promising osmotic agent that simultaneously functions as an osmometabolic agent, providing osmotic drive while improving peritoneal metabolism to potentially mitigate fibrosis. As an essential nutrient for mitochondria, L-carnitine can bind to acyl/acetyl-CoA to form acyl/acetyl-L-carnitine, maintaining the balance between acyl/acetyl-CoA and free CoA. An imbalance in this ratio can impair cellular energy production and metabolic processes [38–44]. Additionally, L-carnitine can relieve the activation of pyruvate dehydrogenase kinase (PDK) by acetyl-CoA, thereby promoting pyruvate oxidation and reducing lactate generation, which is harmful to peritoneal mesothelial cells [45–47].

Gaggiotti et al. [48] systematically evaluated the biocompatibility of 1.5% L-carnitine PDF against glucose PDF and icodextrin through cellular and animal experiments. The results demonstrated that L-carnitine performed much better in terms of cell proliferation capacity, cytotoxicity, ultrastructural integrity, and secretory function. Although icodextrin and 1.5% L-carnitine showed comparable performance in cell proliferation and cytotoxicity, 1.5% L-carnitine exhibited superior protection of microvilli architecture, vacuolization, and cell detachment. Experiments on rabbits further confirmed that 1.5% L-carnitine could effectively preserve the normal structure of the peritoneal mesothelial layer, with reduced submesothelial edema/inflammatory infiltration, and showed no vascular basement membrane thickening.

Collectively, L-carnitine demonstrates unique dual osmometabolic properties that preserve peritoneal structure while suppressing inflammation and vascular pathology, positioning it as a safer long-term PD alternative.

### 4.3. L-carnitine + xylitol

*Xylitol* (151.2 Da), a low-glycemic-index pentose sugar alcohol commonly used as a glucose substitute in foods, has been investigated as a novel osmotic agent in PDF. Masola et.al [49] evaluated XyloCore^™^ (a PDF containing low glucose, xylitol, and L-carnitine) versus glucose PDF in human peritoneal mesothelial cells. *In vitro* analyses demonstrated superior cytoprotection with XyloCore^™^, preserving cell viability, morphology, and barrier function despite repeated exposures. Mechanistically, it attenuated peritoneal fibrosis by suppressing TGF-β/Snail/α-smooth muscle actin (α-SMA) pathway activation, while reducing inflammatory cytokines. Furthermore, Piccapane et al. [50] compared the biocompatibility of XyloCore^™^ with icodextrin and amino acid PDF. The results revealed that the cell viability was similar across all three PDFs, significantly higher than that of the glucose PDF. However, a mild decrease in the tight junction marker Zonula Occludens-1 (ZO-1) was detected in the icodextrin and amino acid PDF groups.

In a Phase II clinical trial [51], Rago et al. explored the safety and efficacy of a novel PDF based on l-carnitine and xylitol. The results demonstrated good safety profiles. However, this study was limited by its small sample size and short observation period. To address these limitations, the research team is conducting a Phase III randomized controlled trial [52]. This trial will comprehensively evaluate the non-inferiority of L-carnitine- and xylitol-based PDF to standard glucose regimens, as well as their long-term metabolic benefits.

### 4.4. Glycerol

*Glycerol* (92 Da) has emerged as a promising osmotic agent because of its favorable biocompatibility and metabolic profile. Early studies demonstrated its dual capacity to provide UF while avoiding the high caloric burden and metabolic complications associated with glucose PDF. Matthys et al. reported stable glycemic control and reduced insulin requirements in patients with diabetes using glycerol PDF, with no significant toxicity during extended use [53]. To further optimize the performance of PDF, researchers have attempted to mix glycerol with traditional osmotic agents such as glucose and amino acids. The GLAD formulation (1.4% glycerol/0.5% amino acid/1.1% dextrose) preserved peritoneal integrity in animal models, which was significantly superior to glucose PDF [54]. Similarly, a clinical trial by Van Biesen et al. demonstrated the safety and tolerability of 0.6% amino acid and 1.4% glycerol blends, which reduced the glucose load while elevating dialysate CA125 levels (a biomarker of mesothelial cell protection) [55,56]. However, there are some potential issues associated with using glycerol as a PDF, such as hyperosmolar syndrome and elevated plasma triglyceride levels [53]. Although mixed osmotic regimens (e.g. GLAD) alleviate these issues to some extent, further studies are required to determine the optimal glycerol concentration and dosage to balance efficacy and safety.

### 4.5. Other Crystalline osmotic agents

Nishimura et al. [57] tested *taurine* as an osmotic agent on rats. The taurine group exhibited significantly lower proliferation of mesothelial cells and fibroblast-like cells than glucose PDF group. However, the low molecular weight (125 Da) of taurine facilitates its peritoneal absorption. Further studies are required to validate the long-term safety. Taurine exhibits cardiovascular protective effects; however, its biosynthesis is impaired in CKD patients [58]. The use of taurine as an osmotic agent may not only alleviate peritoneal fibrosis but also reduce the occurrence of cardiovascular adverse events [57].

*D-allose* (an epimer of D-glucose) is remarkably low in calories and nontoxic [59]. D-allose exhibits antioxidant properties that decrease oxidative stress as well as anti-inflammatory effects [59,60]. One study suggested that D-allose improves cognitive function in mice [61]. Leveraging these advantages, Ozaki et al. [62] developed a PDF by partially replacing D-glucose with D-allose. This novel PDF enhances the viability of rat peritoneal mesothelial cells and reduces inflammatory responses. Furthermore, the novel PDF provided superior protection against hyperglycemia in rats compared to glucose PDF.

In summary, novel crystalline osmotic agents have progressively overcome the limitations of glucose PDF through improved biocompatibility, metabolic modulation, and enhanced reparative functions. Table 1 systematically compares key physicochemical properties of traditional and novel crystalline osmotic agents—including molecular weight, pH, advantages, and limitations—thereby establishing a theoretical basis for future clinical translation.

**Table 1.** Crystalline osmotic agents for peritoneal dialysis Fluids (PDF).

Osmotic Agent	MW (Da)	pH	Advantages	Limitations
Glucose	180	4.5–5.5	Low cost and accessibility Predictable osmotic efficacy	Peritoneal inflammation, fibrosis, neovascularization Glycemic fluctuations, insulin resistance Increased cardiovascular risk
pH-neutral, low-GDP Glucose PDF	180	7.0–7.4	Superior biocompatibility to glucose PDF Preserves residual renal function	Problems with glucose as an osmotic agent remain
Amino Acids	75–204	6.0-7.4	Nutritional supplementation for malnourished patients	Metabolic acidosis with prolonged use Gastrointestinal side effects
Taurine	125	7.2	Good biocompatibility	Evidence limited to animal studies Renal clearance-dependent; long-term safety unverified
D-allose + Glucose	180	Neutral	Good biocompatibility	Evidence limited to animal studies Long-term safety unverified
NAG	221	7.03	Good biocompatibility Improves glycemic control	Evidence limited to animal studies Long-term safety unverified
XyloCore^™^ (Xylitol + L-Carnitine)	151.2 / 161.2	Neutral	Good biocompatibility Improves glycemic control	Evidence limited to animal studies Long-term safety unverified
Glycerol	92	Neutral	Good biocompatibility Improves glycemic control	Hyperosmolar syndrome risk Elevated plasma triglycerides

NAG: N-acetylglucosamine.

GDP: Glucose degeneration product.

## 5. Innovations in osmotic agents- colloidal osmotic agents

### 5.1. Steviol glycosides (SG) and rebaudioside a

*SG* and *rebaudioside A* are active compounds extracted from stevia, with molecular weights ranging from 950 to 1,050 Da. They are used as sweeteners alternative to glucose in the food industry [63]. SG possesses antioxidant, anti-inflammatory, and nephroprotective properties as well as improved glucose tolerance and insulin sensitivity [64–68]. Based on these potential benefits, Kopytina et al. [69] developed a novel PDF using SG as an osmotic agent, and both *in vivo* and *in vitro* studies showed that the SG PDF achieved a UF capacity comparable to that of glucose PDF while exhibiting superior biocompatibility.

### 5.2. Hyperbranched polyglycerol (HPG)

HPG is a polyether polymer characterized by its highly branched structure and molecular weight, typically ranging from hundreds to thousands of Daltons. The HPG PDF at various concentrations demonstrated significant UF and effective urea clearance, as well as reduce peritoneal membrane injury [70]. A subsequent study [70] found that HPG within the 1–3 kDa range demonstrated superior urea removal and sustained UF efficacy—significantly longer than that of glucose PDF. Long-term experiments [71] found that prolonged use of HPG PDF did not cause significant structural changes or functional impairment of the peritoneal membrane, and can effectively suppresses inflammatory signaling pathway activation [71].

Recent evidence extends to metabolic syndrome (MetS) models [72]. In a 3-month trial using obese type II diabetic rats, HPG PDF demonstrated superior biocompatibility compared to physiological and icodextrin. HPG preserves peritoneal membrane integrity, reduces submesothelial thickening and UF loss, and minimizes systemic metabolic disturbances. HPG maintained stable glucose levels and showed mild effects on serum albumin and cytokine levels. Importantly, its’ uniquely preserved the antioxidant capacity and attenuated glomerular injury in diabetic kidneys, highlighting its dual protective role in local and distant organ systems. These findings underscore HPG’s potential in addressing the unmet need for MetS-associated renal failure. However, pharmacokinetic study revealed the clearance of small HPG (1 kDa) was significantly delayed in rats with renal insufficiency. In the future, the potential risk of HPG accumulation (particularly 1 kDa) in patients with ESKD should be careful consideration [73].

Beyond icodextrin—already widely adopted clinically—novel colloidal agents such as SG and HPG demonstrate substantial potential in UF durability, biocompatibility, and metabolic protection. The molecular characteristics, advantages, and limitations of these colloidal osmotic agents are consolidated in Table 2, highlighting their development value as dual-function carriers for “fundamental osmotic capacity + sustained UF maintenance”.

**Table 2.** Colloidal osmotic agents for peritoneal dialysis Fluids (PDF).

Osmotic Agent	MW(Da)	pH	Advantages	Limitations
Icodextrin	13k-19k	/	Superior biocompatibility to conventional glucose PDF Sustained UF with long dwell Improves insulin sensitivity	Maltose accumulation risk Pseudo-hyperglycemia Suboptimal UF vs. glucose in short dwell
HPG	1k-3k	/	Excellent biocompatibility Ameliorates systemic metabolic disturbances Reduces oxidative stress; protects residual renal function	Evidence limited to animal studies Long-term safety unverified (esp. low-MW HPG)
SG	0.9k-1.05k	6.17-6.29	Good biocompatibility Anti-inflammatory effects	Evidence limited to animal studies Long-term safety unverified

UF: Ultrafiltration.

HPG: Hyperbranched polyglycerol.

MW: Molecular weight.

SG: Steviol glycosides.

## 6. Advanced buffering systems

In addition to innovations in osmotic agents, alternative buffers have been explored to replace lactate. Lactate remains the predominant buffer used in conventional PDF. However, lactate-containing PDF is harmful to peritoneal mesothelial cells and can exacerbate peritoneal angiogenesis and fibrosis [45]. The development of dual-chamber bag system makes the usage of bicarbonate-buffered system a possible. Although the bicarbonate represents an improvement over lactate buffer, researchers have also begun exploring buffers with peritoneal protective effects.

### 6.1. Citrate

Previous studies indicated that blocking the complement and coagulation system activation during PD could improve UF [74]. *Citrate* can chelate calcium ions and has emerged as the most prevalent anticoagulant agent for continuous renal replacement therapy (CRRT) [75,76]. Citrate also effectively inhibits complement activation, even at low concentrations (0.25 mmol/L), comparable to levels observed during CRRT [77], and has been shown to reduce oxidative stress [78]. In terms of safety, citrate is metabolized primarily in the liver to bicarbonate with calcium release, making it suitable for patients with renal disease.

Cavallini et al. [79] partially replaced lactate in glucose PDF with citrate. Intraperitoneal glucose retention and net UF were significantly higher with 10 and 15 mmol/L citrate, and net UF was positively correlated with the intraperitoneal citrate concentration. Clinical trials have shown that adding 5 mmol/L citrate to a single PD session significantly increases the net UF and improves creatinine/phosphate clearance [80]. This effect is likely mediated by the calcium-chelating properties of citrate, which modulate calcium-dependent cellular processes, such as smooth muscle contraction and integrin-mediated signaling. Although transient plasma free ionized calcium decreases (∼0.04 mmol/L), rapid hepatic metabolism prevents acid-base imbalance or other adverse effects [80]. Long-term studies in rats [81] confirmed that partial lactate replacement with 10 mmol/L citrate significantly prolongs the lifespan of the catheter.

### 6.2. Pyruvate

Westrhenen et al. [82] found that pyruvate-buffered PDF (35 mmol/L) significantly reduced peritoneal neo-angiogenesis and interstitial fibrosis compared to lactate-buffered PDF. Mechanistically, pyruvate may inhibit glucose-driven polyol pathway activation by lowering the intracellular NADH/NAD + ratio (pseudohypoxic state), thereby reducing the production of profibrotic factors.

A subsequent study [82] evaluated a complex pyruvate-buffered PDF (PYRAGG, containing 1.1% glucose, 0.5% amino acids, and 1.4% glycerol). After prolonged exposure, the PYRAGG group showed 59% lower vascular density and less fibrosis than the lactate group. The heat-sterilized lactate group exhibited more severe fibrosis owing to the presence of GDPs. These findings suggest that pyruvate not only attenuates glucose toxicity through metabolic modulation but may also improve long-term peritoneal structural integrity by counteracting GDP-mediated damage.

## 7. Adding peritoneal protective substances

In addition to osmotic agents and buffers, incorporating specific additives into PDF represents another strategy to protect the peritoneum. The efficacy of these additives is often measured by their ability to modulate key signaling pathways in fibrosis. Pro-fibrotic mediators such as VEGF, connective tissue growth factor (CTGF), TGF-β and α-SMA drive angiogenesis and tissue fibrosis. Conversely, bone morphogenetic protein 7 (BMP-7) and inhibitory Smad proteins help counteract fibrotic processes. The following substances confer protection by positively influencing this balance.

### 7.1. Glycosaminoglycans (GAGs)

GAGs are the key components of extracellular matrix. They bind large amounts of water to form hydrated gels, maintain tissue elasticity, and provide cushioning. GAGs encompass a broad group of substances including chondroitin sulfate, dermatan sulfate, heparan sulfate, and HA.

#### 7.1.1. Sulodexide

*Sulodexide* consists of 80% fast-mobility heparin and 20% dermatan sulfate, is mainly used to treat thrombotic and peripheral vascular diseases. Bazzato et al. [83] conducted an intervention by adding 50 mg of sulodexide nightly to patients’ PDF. The results demonstrated significant increases in dialysate-to-plasma ratios for urea and creatinine compared with baseline. In addition, the protein levels in effluent significantly decreased, while serum albumin concentrations increased. Sulodexide also reduced peritoneal inflammation. Fracasso et al. [84] observed a substantial reduction in inflammatory cytokines within the effluent of six PD patients after 5 months of oral sulodexide. Misian et al. [85] further validated the anti-inflammatory and peritoneal protective effects of sulodexide. They cultured peritoneal mesothelial cells with sulodexide added to glucose PDF, which significantly reduced intracellular free radical generation and suppressed pro-inflammatory, profibrotic, and pro-angiogenic gene expression.

#### 7.1.2. Hyaluronic acid (HA)

*HA* is a major extracellular matrix component that is synthesized by mesothelial cells. It contributes to re-mesothelialization, maintains cell phenotype, facilitates tissue remodeling, and provides structural support to the peritoneal membrane [86,87].

Wang et al. [88] found that the addition of 0.01% HA to glucose PDF significantly increased the net UF volume in rats. Subsequent findings indicated that 500k Da molecular weight HA was most effective at reducing PDF absorption and increasing net UF, likely by decreasing tissue hydraulic conductivity and increasing fluid viscosity [89,90]. Breborowicz et al. [91] and Połubinska et al. [92] reported that HA supplementation reduced transperitoneal albumin loss, increased UF, and diminished peritoneal inflammation in rats. In rat model of PD with induced acute colitis, supplementation of HA markedly attenuated the pronounced intra-abdominal inflammatory response [93].

However, emerging evidence suggests that HA paradoxically exacerbates peritoneal inflammation and fibrosis under specific conditions [86,87]. Although HA promotes tissue repair and mitigates acute inflammation, sustained overexpression or abnormal submesothelial HA accumulation may drive pathological processes. High-molecular-weight HA can induce Snail2 expression in fibroblasts, which is a key mechanism in peritoneal fibrosis [94]. In keratocytes, HA accumulation mediates TGF-β1-induced α-SMA and fibronectin synthesis, both of which are hallmarks of fibrotic remodeling [95]. These findings imply that while HA supplementation may transiently mitigate inflammation and improve UF in acute settings, its chronic overexpression, could tip the balance toward maladaptive fibrosis and sustained peritoneal damage. Future experiments are needed to prove the long-term effects of HA on peritoneal morphology.

#### 7.1.3. Low-molecular-weight heparins (LMWH)

Both *LMWH* and HA belong to the glycosaminoglycan family. In addition to HA, researchers have attempted to add LMWH to the PDF to alleviate peritoneal inflammation and fibrosis. Clinical studies have demonstrated that intraperitoneal administration of LMWH significantly reduced dialysate-to-plasma ratios of creatinine, urea, and albumin, while enhancing UF, without increasing the risk of bleeding [96]. Researchers further revealed that the addition of LMWH to PDF can suppress systemic inflammatory responses and intraperitoneal interleukin-6 (IL-6) production, ameliorating chronic inflammation in PD patients [97]. Mechanistic insights from animal models have highlighted LMWH’s dual role: it inhibits thrombin-antithrombin complex formation and C5a-dependent chemotactic activity, thereby reducing coagulation and neutrophil recruitment [98]. Additionally, LMWH downregulated hypoxia-inducible factor 1-α, VEGF, and TGF-β1 expression in a dose-dependent manner [99]. Collectively, these findings underscore LMWH’s multi-target modulation of the inflammatory, coagulative, and fibrotic pathways, offering a promising therapeutic approach for preserving peritoneal membrane integrity.

### 7.2. Molecular hydrogen (H_2_)

In recent years, H_2_ received a great deal of attention in the medical field because of its unique antioxidant, anti-inflammatory, and cytoprotective properties. Studies have shown that H_2_ can selectively remove hydroxyl radicals and peroxynitrite, and modulate critical signaling pathways, which alleviates oxidative stress and inflammatory damage in various disease models [100,101].

Recent animal and clinical studies demonstrated the potential benefits of incorporating H_2_ into PDF. Terawaki et al. [102] demonstrated that H_2_ PDF significantly reduced peritoneal and systemic oxidative stress. Another study by Nakayama et al. [103] found that H_2_ PDF preserved the integrity of mesothelial cells and the peritoneal membrane in rats, with a significant reduction in submesothelial thickening and mesothelial cell loss. Furthermore, H_2_ treatment was associated with a shift toward the M2 macrophage phenotype, which is known for its tissue-repairing properties. In addition to these findings, a small clinical trial demonstrated the feasibility and safety of H_2_ PDF [104]. This study also observed increased levels of CA125 and mesothelin in the effluent, suggesting enhanced mesothelial regeneration [56,105]. Lu et al. [106] revealed that H_2_ alleviates peritoneal fibrosis by scavenging reactive oxygen species (ROS) and inhibiting the phosphatase and tensin homolog (PTEN)–protein kinase B–mechanistic target of rapamycin (mTOR) signaling pathway.

Overall, these studies confirmed the safety of H_2_ PDF and suggested that it may delay the progression of peritoneal fibrosis and promote mesothelial regeneration, which could improve the long-term outcomes of PD treatment. Future studies should focus on large-scale clinical trials to explore the potential value of H_2_ PDF in improving patient prognosis and reducing PD-related complications.

### 7.3. Alanyl-Glutamine (Ala-gln)

As a cytoprotective agent, *Ala-Gln* can protect peritoneal mesothelial and endothelial cell functions. Basic studies demonstrated that Ala-Gln upregulates claudin-5 and ZO-1 expression, restores transendothelial electrical resistance, and reduces dextran permeation, suggesting tight junction remodeling [107]. Furthermore, Ala-Gln was found to correct the function of 378 proteins, including cell junction and cytoskeletal-related proteins [108]. In rat and mouse studies, Ala-Gln PDF reduced peritoneal thickness and the density of α-SMA-positive cells. This effect is potentially mediated through suppression of TGF-β-driven collagen deposition *via* regulation of Th17 differentiation and IL-17 signaling, thereby ameliorating PD-induced injury [109]. Collectively, these findings indicate that Ala-Gln enhances peritoneal barrier repair and permeability through multiple target mechanisms.

A preliminary clinical trial suggested that Ala-Gln could enhance cellular self-healing, and reduce peritoneal aseptic inflammation. In patients with peritonitis, Ala-Gln-enriched PDF can reduce IL-8 concentration in the effluent. These findings offer preliminary evidence for the anti-inflammatory effects of Ala-Gln in the human peritoneum [110]. Vychytil et al. conducted a clinical trial [111] and reported a significant increase in CA-125 levels in the effluent, along with reduced transperitoneal protein loss and lower systemic inflammatory markers.

A targeted metabolomics study revealed that Ala-Gln decreased oxidative stress markers in effluent, suggesting attenuation of oxidative damage [112]. A randomized trial revealed that Ala-Gln enhances peritoneal immune cell responsiveness, as evidenced by significantly increased IL-6 and TNF-α release following *ex vivo* stimulation [113]. Transcriptome analysis revealed that Ala-Gln modulates the expression of genes associated with immune function and inflammation, suggesting its immunomodulatory properties. However, further research is required to ascertain its potential for reducing infectious complications.

Collectively, these additives demonstrate multi-target modulation of inflammatory and fibrotic pathways. Table 3 provides a comparative overview of peritoneal protective additives—including HA, sulodexide, LMWH, H_2_, and Ala-Gln—summarizing their advantages and limitations to inform clinical translation strategies.

**Table 3.** Peritoneal protection additives in peritoneal dialysis Fluids (PDF).

Additive	Advantages	Limitations
HA	Increases net UF Reduces transperitoneal albumin loss Alleviates peritoneal inflammation	Evidence from animal studies only May exacerbate peritoneal fibrosis at high dose
Sulodexide	Improves dialysis efficiency (creatinine/urea clearance) Reduces transperitoneal albumin loss Suppresses peritoneal inflammation and oxidative stress	Evidence from small-scale clinical trials
LMWH	Enhances net UF Reduces transperitoneal albumin loss	Decreases dialysis efficiency (creatinine/urea clearance) Evidence from animal studies and small clinical trials
H₂	Alleviates peritoneal and systemic inflammation Alleviates peritoneal fibrosis Promotes mesothelial regeneration	Evidence from animal studies and small clinical trials
Ala-Gln	Alleviates peritoneal inflammation and oxidative stress Promotes peritoneal barrier repair Enhances peritoneal immune cell responsiveness	Evidence from animal studies and small clinical trials (including RCTs)

HA: Hyaluronic acid.

UF: Ultrafiltration.

LMWH: Low molecular weight heparin.

H_2_: Molecular hydrogen.

Ala-Gln: Alanyl-glutamine.

## 8. Improvement of UF and treat of heart failure

In addition to its role in RRT for patients with ESKD, the UF capability of PDF is gaining attention for managing volume overload. Refractory congestive heart failure (RCHF) poses significant clinical challenges with a suboptimal prognosis. Studies indicate that over 50% of RCHF patients develop cardiorenal syndrome, which is associated with elevated mortality risks [114]. Peritoneal UF has emerged as a novel therapeutic strategy for RCHF, showing potential benefits in varies studies [115–122]. However, current evidence primarily stems from small sample-observational studies, predominantly using glucose PDF, which carries the risk of metabolic complications and peritoneal damage. Prior studies suggest that novel PDFs, such as PolyCore^™^, offer favorable biocompatibility and UF efficacy comparable to that of glucose. To further evaluate UF in RCHF, Gronda et al. [123] designed a multicenter randomized controlled trial to evaluate the safety and efficacy of PolyCore^™^. However, the study is ongoing, and no specific results are available.

To address the severe sodium and water retention issues in patients with edematous disorders, such as heart failure, Asher et al. [124] developed a novel PDF containing 30% icodextrin and 10% dextrose. This formulation significantly increased the UF volume in rats. Subsequent validation in sheep confirmed the enhanced sodium and water removal capacity, achieving approximately 3.5 times greater UF and 4 times greater sodium removal compared to 7.5% icodextrin. Long-term biocompatibility studies in mice and sheep showed no significant increase in peritoneal damage compared with 4.25% glucose PDF. A small clinical study found this novel PDF achieved good clearance of sodium and water, and no serious adverse events occurred.

Leypoldt et al. [125] explored low-polydispersity glucose polymers to enhance UF efficiency without increasing carbohydrate absorption (CA). Using a computer-simulated three-pore model, they predicted UF and CA for polymers with different molecular weights, polydispersity, and concentrations. The results showed that polymers with high molecular weight (≥7.5 kDa) and low polydispersity at higher concentrations may improve UF without increasing CA. Experiments in rabbits have confirmed this hypothesis. In contrast, increasing the concentration of icodextrin to 11% significantly increased the CA without improving UF, highlighting the advantages of low-polydispersity polymers [125].

Beyond osmotic agent modifications, dialysis modality innovations aim to enhance UF. Heimürger et al. [126] evaluated steady-state peritoneal dialysis (SCPD) in a preliminary crossover trial (*n* = 8). Compared to the standard 4-h PD with 2.5% glucose, SCPD significantly increased the UF rate. Sodium removal and glucose UF efficiency (UF volume per gram of absorbed glucose) were also significantly higher in SCPD. Wilkie et al. [127] further investigated the efficacy and safety of SCPD in home setting. This prospective multicenter study (*n* = 19) confirmed its advantages in enhancing UF, increasing sodium removal, and improving glucose UF efficiency.

## 9. Conclusions

Current research and development of novel PDF primarily aims to overcome the dual limitations of traditional glucose PDF—biological incompatibility and metabolic complications. This review synthesizes recent advancements in biocompatible PDF, charting a course beyond the limitations of glucose PDF. The future of PDF development lies in the strategic integration of three key components: (1) novel osmotic agents (e.g. L-carnitine, HPG, NAG) that mitigate fibrosis and metabolic side effects; (2) advanced buffers (e.g. citrate, pyruvate) that enhance biocompatibility and UF; and (3) peritoneal protectants (e.g. Ala-Gln, H_2_) that actively counter inflammation and fibrosis.

While previous reviews have outlined some novel biocompatible agents in PDF [128,129], our work provides an update and expansion. This review synthesizes a broader spectrum of novel agents, including advanced buffers and more osmotic agents and introduce the forward-looking concept of synergistic “osmotic agent + buffer + additive” formulations. Furthermore, beyond the progress in biocompatible formulation, this review also focuses on the emerging application in managing RCHF, thereby expanding the therapeutic scope of PD beyond traditional renal replacement therapy.

However, this study also has limitations. The current evidence is largely based on animal studies and small-scale clinical trials. Future large-scale clinical research is needed to validate the long-term safety, efficacy and pharmacokinetics of these novel osmotic agents, buffers, and additives. As an integrative review, our analysis is also inherently influenced by the availability and quality of the existing literature, and our novel conceptual framework, while informed by the evidence, awaits formal clinical validation.

## Data Availability

Not available.
